# 新型免疫肿瘤治疗临床研究现状及应用前景

**DOI:** 10.3779/j.issn.1009-3419.2017.09.10

**Published:** 2017-09-20

**Authors:** 世佳 张, 胜祥 任

**Affiliations:** 200433 上海，同济大学附属上海市肺科医院肿瘤科 Department of Medical Oncology, Shanghai Pulmonary Hospital, Tongji University School of Medicine, Shanghai 200433, China

**Keywords:** 肿瘤, 免疫肿瘤治疗, 免疫检查点抑制剂, Neoplasms, Immuno-oncology therapy, Immune checkpoint inhibitor

## Abstract

近年来，免疫肿瘤治疗发展迅速，已经成为继手术、放疗、化疗、靶向治疗后癌症的另一有效治疗手段。目前已有多个免疫检查点相关药物获批用于多种恶性肿瘤的治疗，而且越来越多的潜在治疗靶点受到关注。本综述旨在根据肿瘤-免疫循环中的不同环节，对不同机制的免疫肿瘤治疗药物研究现状和临床前景进行概述和展望。

## Abstract (en)

Recently, immune-oncologic therapy advanced rapidly and has been defined as another option, following surgery, radiotherapy, chemotherapy and molecular targeted therapy, for treatment of malignant diseases. To date, several immune checkpoint inhibitors and compounds have been approved to treat various of malignant diseases with efficiency. Meanwhile, more and more potential therapeutic targets in processes of the cancer immunity have been noticed. We aimed to summarize the research status and clinical prospects of novel immune-oncologic treatment agencies targeted to different steps of the cancer-immunity cycle.

人免疫系统可对抗肿瘤这一概念的形成最早可追溯至19世纪50年代，德国病理学家Rudolf Virchow首次观察到人肿瘤组织中的免疫细胞浸润。其后，美国外科医生Coley首次采用在肿瘤软组织中注射细菌的方法诱导免疫应答^[1]^。近年来，随着抗体制备等相关技术的成熟，免疫肿瘤（immuno-oncology, I-O）治疗发展迅速，已经成为继手术、放疗、化疗、靶向治疗后癌症的另一有效治疗手段。尤其是明确了导致T细胞免疫反应抑制的“免疫检查点”机制后，已有多个免疫检查点相关药物获美国食品和药品监督管理局（Food and Drug Administration, FDA）批准用于多种恶性肿瘤的治疗^[2]^。随着对肿瘤免疫机制的进一步明确，越来越多抑制和活化通路上的关键位点受到关注，研究者对针对这些位点的药物进行了大量的临床前和临床研究。本文就下一代免疫肿瘤治疗相关研究进行概述和展望如下。

## 1 肿瘤免疫机制和I-O治疗的基础

### 1.1 肿瘤免疫机制

2013年，Chen和Mellman^[3]^提出“肿瘤-免疫循环”的概念，即抗肿瘤免疫反应有效杀伤肿瘤细胞，需要经历数个步骤：①肿瘤形成后生成新抗原，释放入机体后被树突状细胞（dendritic cell, DC）捕获；②DC将捕获的抗原递呈至T细胞；③效应T细胞激活，产生对抗癌症抗原的特异性免疫反应（在这一阶段确定免疫反应的性质，效应T细胞和调节T细胞的比例是最终结局的重要预测因素）；④活化的效应T细胞迁移至肿瘤床；⑤活化的效应T细胞在肿瘤床浸润；⑥效应T细胞特异性识别并与肿瘤细胞结合（通过同源抗体与组织相容性复合物I结合）；⑦目标肿瘤细胞被杀灭；⑧被杀灭的肿瘤细胞进一步释放更多肿瘤相关抗体（即回到步骤①）。通过这一循环使免疫反应范围增大，强度增强。但在恶性肿瘤患者中，DC和T细胞可能将肿瘤抗原视为自体成分而不被识别，T细胞无法在肿瘤组织中浸润，或肿瘤微环境中的一些因素抑制效应T细胞的功能，这些因素均会导致肿瘤-免疫肿瘤循环应答无法达到最佳效果。

I-O治疗的目标是启动或重启患者的癌症-肿瘤循环，放大免疫效应，但不造成无限制的自身免疫应答。最有效的I-O治疗方案可能是选择性针对每例患者的限速步骤进行治疗。近期研究提示肿瘤微环境导致的免疫抑制是常见的限速步骤^[3]^。

### 1.2 I-O治疗的基础

I-O治疗通常根据其是否具有激活宿主免疫系统抗肿瘤细胞的能力，被分为“主动免疫”和“被动免疫”两大类^[4]^。免疫检查点抑制剂通过阻断肿瘤微环境中的免疫抑制因素；肿瘤特异性活化通路激动剂、细胞因子等通过活化宿主的抗肿瘤免疫系统，旨在恢复或提高宿主机体的抗肿瘤免疫作用，因此属于主动免疫。而过继T细胞治疗、肿瘤特异性单克隆抗体等治疗方法将本身有免疫功能的成分输入机体内，属于被动免疫。

### 1.3 I-O治疗的特征

I-O治疗带来的临床获益具有一系列其他抗肿瘤治疗中从未观察到的特征，其中最主要的特征是I-O治疗能带来持久获益，能给患者带来前所未有的长期生存。长期随访研究结果显示，既往接受过多线治疗的非小细胞肺癌（non-small cell lung cancer, NSCLC）患者接受nivolumab治疗后，5年生存率为16%，提示总生存期（overall survival, OS）OS获益具有长期持续的特点^[5]^。在比较T细胞免疫检查点PD-1抑制剂nivolumab与依维莫司用于转移性肾癌患者的Ⅲ期临床试验中，虽然两组中位无进展生存（progression-free survival, PFS）时间无差异，但nivolumab组中位OS明显延长（25个月*vs* 19.6个月；*P*≤0.014, 8）^[6]^。而且即使由于毒性反应等原因停用免疫检查点阻断治疗，治疗反应仍可维持^[7]^。此外，接受I-O治疗的患者可能出现假性进展——定义为肿瘤并未进一步生长，但由于出现免疫细胞浸润，因此在影像学上观察到肿瘤体积增大。在接受nivolumab和另一种PD-1抑制剂pembrolizumab治疗的黑色素瘤患者中，分别有8%和15%的患者发生假性进展^[8]^，因此应采用免疫相关疗效评价标准（immune-related response criteria, irRC）评估疗效^[9]^。I-O治疗相关不良事件也有一定的特征性，常见免疫相关不良事件包括ALT升高、结肠炎、甲状腺功能亢进和减退、垂体炎和肺炎等，早期诊断和及时应用类固醇治疗是管理这类不良事件的关键^[10]^。

## 2 I-O治疗药物的研究现状

### 2.1 效应T细胞机制

#### 2.1.1 抑制通路

CTLA-4：细胞毒T淋巴细胞相关抗原4（cytotoxic T-lymphocyte associated protein 4, CTLA-4）是研究最深入的免疫检查点受体之一。CTLA-4表达于T细胞和调节T细胞表面。肿瘤细胞利用CTLA-4通路抑制免疫应答的产生，并减弱T细胞活性及其增殖为记忆T细胞的能力。使用CTLA-4特异性抗体治疗能增加记忆T细胞的累积和存活，并促进调节T细胞的耗竭，从而使免疫应答功能恢复^[11]^。CTLA-4抑制剂伊匹木单抗（ipilimumab）2011年获美国FDA批准用于无法切除或转移性黑色素瘤的治疗，2015年先后获批用于有局部淋巴瘤浸润或直径 > 1 mm的皮肤黑色素瘤手术后患者的辅助治疗，以及与PD-1抑制剂nivolumab联合治疗BRAF V600野生型的无法切除或转移性黑色素瘤^[2]^。目前，多项伊匹木单抗联合肿瘤疫苗、程序性死亡因子1（programmed cell death protein 1, PD-1）抑制剂、肿瘤靶向药物等用于各种晚期实体瘤和血液学恶性肿瘤的Ⅰ期-Ⅲ期临床试验正在进行中。其他CTLA-4通路药物包括AGEN-1884和tremelimumab等也有一些Ⅰ期-Ⅱ期临床试验正在开展（表 1）。

**1:** 靶向CTLA-4和PD-1的药物和正在进行的临床试验 Agents approved and in clinical trial targeting CTLA-4 andPD-1 pathways

Generic name	Trade name	Manufacturer	Target	Indications (approved year)	Conditions in clinical trials
Ipilimumab	Yervoy	Bristol-Myers Squibb	CTLA-4	Unresectable or metastatic melanoma (2011); adjuvant treatment of patients with cutaneous melanoma with pathologic involvement of regional lymph nodes who have undergone complete resection (2015)	Hepatocellular carcinoma, lung cancer, pancreatic cancer, breast cancer, metastatic brain tumor, renal cell carcinoma, prostate cancer, human immunodeficiency virus-related solid tumors, leukemia, lymphoma, other rare and refractorytumors
AGEN-1884	-	Agenus, Inc.	CTLA-4	-	Advanced solid tumors
Tremelimumab	-	AstraZeneca	CTLA-4	-	Lung cancer, hepatocellular carcinoma, colorectal cancer, pancreatic cancer, esophageal cancer, head and neck cancer, breast cancer, ovarian cancer, renal cell carcinoma, lymphoma, melanoma
Nivolumab	Opdivo	Bristol-Myers Squibb	PD-1	Unresectable or metastatic melanoma (2015); metastatic non-small cell lung cancer (2015); advanced renal cell carcinoma (2015); Hodgkin lymphoma (2016); recurrence or metastatic head and neck squamous cell carcinoma (2016); advanced or metastatic urothelial carcinoma (2017)	Colorectal cancer, prostate cancer, hepatocellular carcinoma
Pembrolizumab	Keytruda	Merck	PD-1	Unresectable or metastatic melanoma (2014-2015); non-small cell lung cancer (2015); recurrent or metastatic head and neck squamous cell carcinoma (2016); refractory Hodgkin lymphoma (2017); advanced or metastatic urothelial carcinoma (2017); microsatellite instability-high or mismatch repair deficient solid tumors (2017)	Advanced refractory malignant solid tumors, hematological malignancies
AMP-224	–	GlaxoSmithKline	PD-1	–	Colorectal cancer
AMP-514	–	GlaxoSmithKline	PD-1	–	Invasive B cell lymphoma
Pidilizumab	–	CureTech	PD-1	–	Diffuse large B-cell lymphoma, follicular lymphoma, multiple myeloma
BGB-A317	–	BeiGene, China	PD-1	–	Advanced cancer
SHR-1210	–	Jiangsu Hengrui Medicine	PD-1	–	Advanced solid tumors
BMS-936559	–	Bristol-Myers Squibb	PD-L1	–	Advanced solid tumors, severe sepsis, human immunodeficiency virus infection
Atezolizumab	–	Genentech/Roche	PD-L1	Locally advanced or metastatic urothelial carcinoma (2016); metastatic non-small cell lung cancer (2016)	*Melanoma, renal cell carcinoma, breast cancer, multiple myeloma, * *etc*
MEDI4736	–	AstraZeneca	PD-L1	–	Non-small cell lung cancer, advanced solid tumors
Avelumab	–	Pfizer/Merck	PD-L1	Advanced or metastatic urothelial carcinoma (2017); metastatic Merkel cell carcinoma (2017)	Advanced solid tumors, renal cell carcinoma
rHIgM12B7	–	Mayo Clinic/NCI	PD-L2	–	Metastatic melanoma

PD-1：PD-1是表达于细胞毒T细胞表面的免疫检查点受体，其配体包括PD-L1和PD-L2。临床前研究显示，阻断PD-1可使被耗竭的T细胞复苏，并恢复其细胞毒功能^[12]^。此外，与单纯抑制PD-L1相比，同时抑制PD-L1和PD-L2以完全抑制PD-1信号转导可能更有效地逆转T细胞耗竭。在美国FDA获批的PD-1抑制剂包括nivolumab（黑色素瘤、NSCLC、霍奇金淋巴瘤、头颈部鳞癌、肾细胞癌、尿路上皮癌）、pembrolizumab[黑色素瘤、NSCLC、头颈部鳞癌、霍奇金淋巴瘤、尿路上皮癌和高微卫星不稳定性（microsatellite instability-high, MSI-H）/错配修复缺陷（mismatch repair deficient, dMMR）实体瘤]，PD-L1抑制剂包括atezolizumab（尿路上皮癌和NSCLC）和avelumab（Merkel细胞癌和尿路上皮癌）^[2]^。一些研究显示，肿瘤组织中的PD-L1表达水平与PD-1/PD-L1抑制剂的治疗效果相关^[13-15]^，但还有一些研究得出不同的结论^[6]^。互相矛盾的结果可能与PD-L1检测方法的缺陷有关（缺乏检测金标准、PD-L1在肿瘤中呈群集性表达导致活检易出现假阴性等）^[16]^。其他生物标志物还包括dMMR和肿瘤突变负荷等。Ⅱ期临床试验显示，dMMR结直肠癌、错配修复功能完好（proficient mismatch repair, pMMR）结直肠癌和dMMR非结直肠癌患者接受pembrolizumab治疗后，免疫相关客观缓解率分别为40%、0和71%，免疫相关PFS率分别为78%、11%和57%^[17]^。其他正在进行临床试验的PD-1/PD-L1抑制剂包括AMP-224、AMP-514、pidilizumab、BGB-A317、SHR-1210；PD-L1和PD-L2抑制剂包括BMS-936559、MEDI4736和rHIgM12B7等（表 1）。

LAG-3：淋巴细胞活化基因3（lymphocyte activation gene 3, LAG-3）是表达于活化的细胞毒T细胞和调节T细胞表面的免疫检查点受体。在活化的细胞毒T细胞与肿瘤抗原接触后，LAG-3的数量和活性可稳定升高，导致T细胞耗竭。表达LAG-3的调节T细胞也聚集在肿瘤病灶，且对细胞毒T细胞具有强抑制性。临床前研究表明LAG-3的失活可提高细胞毒T细胞对抗肿瘤的活性^[18]^。早期开发的LAG-3通路药物主要是LAG-3免疫球蛋白IMP321，已在晚期肾细胞癌、胰腺癌、黑色素瘤和乳腺癌中完成Ⅰ期临床试验，显示了一定的抗肿瘤和增强免疫的效果^[19-21]^。此外目前还有几种LAG-3单克隆抗体（BMS-986016、LAG525）正在晚期实体瘤和血液学恶性肿瘤患者中开展单药或与PD-1抑制剂联用的Ⅰ期和Ⅱ期临床试验（NCT01968109, NCT02061761, NCT02750514, NCT02658981, NCT02460224）。

TIGIT：T细胞免疫球蛋白和ITIM结构域（T-cell immunoglobulin and ITIM domain, TIGIT）在NK细胞、效应和记忆T细胞、调节T细胞上均有表达^[18]^。TIGIT与其配体——抗原递呈细胞上的CD155和T细胞上的CD112结合后，竞争性抑制CD226与相同配体结合，而后者可增强细胞毒作用和抗肿瘤免疫应答。临床前研究显示，同时阻断TIGIT和PD-L1可有协同效应，使耗竭的细胞毒T细胞恢复功能，发挥持续的抗原特异性抗肿瘤效应^[22, 23]^。

#### 2.1.2 活化通路

表达于T细胞和其他免疫细胞表面的一系列活化受体被命名为共刺激因子，包括表达于静息淋巴细胞上的受体（如CD27），也包括在接触抗原后才诱导表达的受体[如CD137、糖皮质激素诱导肿瘤坏死因子受体（glucocorticoid-induced tumour necrosis factor receptor, GITR）和OX40]。这些共刺激因子在T细胞的增殖、存活和免疫功能调节方面发挥作用^[24]^。临床前研究显示，活化CD27、CD137、GITR、OX40等信号通路可以增强肿瘤特异性T细胞免疫应答，通过维持细胞毒T细胞的生存和减弱调节T细胞的免疫抑制作用等机制长期维持免疫效果^[25-28]^。

已完成的Ⅰ期临床研究显示，使用鼠源性OX40激活剂型单克隆抗体9B12治疗晚期实体瘤的耐受性较好，可提高外周血CD4和CD8阳性T细胞的增殖水平，改善肿瘤特异性免疫应答，30%（12/30）的患者至少一处转移灶有所好转^[29]^。另一种激活剂型抗体PF-04518600 Ⅰ期临床试验的初步结果也显示，该抗体可有效结合目标受体并增强记忆T细胞的增殖^[30]^。

除单药治疗外，共刺激单克隆抗体联合其他免疫检查点阻断治疗也有良好的应用前景，联合抗表皮生长因子受体（epidermal growth factor receptor, EGFR）、抗人类表皮生长因子受体2（human epidermal growth factor receptor 2, HER2）或抗CD20也有一定的临床前研究结果支持。多种针对这些活化通路的药物，如CD27单克隆抗体varlilumab（NCT02413827, NCT02386111, NCT02335918, NCT02270372）、CD137单克隆抗体IgG4 urelumab（NCT01775631, NCT02110082，NCT02252263, NCT02420938, NCT02253992）和CD137单克隆抗体IgG2 PF05082566（NCT02179918）、GITR激动剂GWN323（NCT02740270）和OX40通路激动剂[包括9B12（NCT02205333）、PF-04518600（NCT02315066）、MEDI0562（NCT02705482）和MOXR0916（NCT02410512）]，联合其他药物的方案目前正在晚期实体瘤和血液恶性肿瘤患者中进行Ⅰ期和Ⅱ期临床试验。

### 2.2 NK细胞机制

NK细胞是免疫系统中迅速响应和机体防御癌症的第一道防线，NK细胞可通过分泌细胞因子/趋化因子进行肿瘤的免疫监视和清除。目前研究较多的活化和抑制通路包括信号淋巴细胞激活分子家族成员7（signaling lymphocytic activation molecule family member 7, SLAMF7）和杀伤细胞免疫球蛋白样受体（killer-cell immunoglobulin-like receptor, KIR）。SLAMF7与配体结合可激活NK细胞，临床前研究显示，SLAMF7的人源化单克隆抗体elotuzumab可不依赖抗体依赖的细胞毒作用（antibody dependent cell mediated cytotoxicity, ADCC）途径直接增强NK细胞的抗肿瘤细胞毒作用^[31]^。Ⅲ期临床试验显示，elotuzumab联合来那度胺和地塞米松用于多发性骨髓瘤患者的治疗，总缓解率高于仅来那度胺和地塞米松组（79% *vs* 66%, *P* < 0.001）^[32]^。KIR是表达于NK细胞表面的免疫检查点受体，抑制KIR可以保护正常细胞免受NK细胞杀伤，而肿瘤细胞可以通过破坏这一过程以逃脱NK细胞介导的识别和摧毁^[33]^。抗KIR2DL单克隆抗体lirilumab目前正在晚期实体瘤和血液恶性肿瘤患者中进行Ⅰ期和Ⅱ期临床试验（NCT01687387, NCT01714739, NCT01592370, NCT02252263, NCT02399917, NCT02481297, NCT02599649）。此外，各种类型或分期的皮肤T细胞淋巴瘤患者均表达KIR3DL2，另一种抗KIR3DL2单克隆抗体IPH4102正在此类患者中进行Ⅰ期临床试验（NCT02593045）。

### 2.3 其他与肿瘤免疫逃逸相关的非效应细胞机制

其他非效应细胞机制包括与调节T细胞相关的CCR4、CD73、吲哚胺-2, 3-双加氧酶-1（indoleamine 2, 3-dioxygenase, IDO）和转化生长因子受体（transforming growth factor receptor, TGFR），以及与肿瘤相关巨噬细胞（tumor-associated macrophage, TAM）相关的集落刺激因子1受体（colony stimulating factor 1 receptor, CSF1R）^[34-36]^。对这些途径进行阻断可减少调节T细胞的数量，减弱其免疫抑制功能，促进效应T细胞和NK细胞的增殖，改善T细胞应答^[37-42]^。人源化抗CCR4单克隆抗体mogamulizumab于2012年在日本获批用于成人T细胞淋巴瘤的治疗^[34]^。其他药物包括CD73非竞争性单克隆抗体MEDI9447（NCT02503774）；IDO抑制剂indoximod（NCT02073123, NCT02460367）、epacadostat（NCT02178722, NCT02318277, NCT02298153, NCT01604889）和GDC-0919（NCT02471846, NCT02048709）；TGFR抑制剂galunisertib（NCT01246986, NCT01220271, NCT02178358）、TEW-7197（NCT02160106）、PF-03446962（NCT02116894）和IMC-TR1（NCT01646203）；CSF-1R小分子抑制剂pexidartinib（NCT02777710, NCT02452424）、BLZ945（NCT02829723）和CSF-1R单克隆抗体LY3022855（NCT02718911）、FPA008（NCT02526017）、IMC-CS4（NCT01346358）等，目前在晚期实体瘤和血液肿瘤患者中进行单药或与其他免疫肿瘤联合治疗（如PD-1抑制剂）的Ⅰ期或Ⅱ期临床试验。

### 2.4 肿瘤细胞靶向通路

ADCC是抗肿瘤免疫中的重要一环。通过ADCC杀伤肿瘤的抗体的Fab段特异结合肿瘤细胞表面抗原，而Fc段与巨噬细胞、NK细胞和中性粒细胞等Fc受体结合，刺激这些细胞释放多种效应分子（如TNF等）杀伤肿瘤细胞。ADCC对肿瘤细胞的杀伤仅需要较少的抗体分子，制备这类抗体进行被动免疫可阻止肿瘤生长。

目前研究较多的治疗性肿瘤靶向抗体包括抗HER2、EGFR、Fucosyl-GM1和趋化因子受体4（chemokine receptor type 4, CXCR4）单克隆抗体。HER2高表达可见于乳腺癌和部分胃癌的肿瘤细胞表面。HER2单克隆抗体曲妥珠单抗已获批准用于转移性乳腺癌、转移性胃癌和胃食管结合部腺癌的治疗。此外，Ⅲ期研究^[43]^显示，帕妥珠单抗和曲妥珠单抗“双重阻断”HER2并联合化疗的方案用于HER2阳性早期乳腺癌患者的新辅助治疗，可获得较高的病理学缓解率。其他抗HER2单克隆抗体还包括margetuximab，目前正在晚期乳腺癌患者中开展Ⅰ期-Ⅲ期临床试验（NCT01828021, NCT02492711）。较早获批的EGFR单克隆抗体包括西妥昔单抗和帕尼单抗，适应证包括头颈部鳞癌和转移性结直肠癌。2015年底，一种新型EGFR单克隆抗体获批与吉西他滨和顺铂联合用于转移性肺鳞癌患者的一线治疗。Fucosyl-GM1在小细胞肺癌细胞表面高表达，其单克隆抗体BMS-986012目前正在小细胞肺癌患者中开展Ⅰ期和Ⅱ期临床试验（NCT02247349, NCT02815592）。CXCR4是肿瘤最常表达的趋化因子受体之一，在肿瘤细胞增殖、迁移、转移、侵袭和存活中均发挥重要作用^[44]^。抗CXCR4单克隆抗体ulocuplumab目前正在晚期实体瘤患者中开展Ⅰ期和Ⅱ期临床试验（NCT02472977, NCT02305563）。

抗体-药物偶联物（antibody-drug conjugate, ADC）是I-O治疗中的另一种思路。ADC由肿瘤细胞靶点特异性结合抗体、稳定的连接体和药物制剂三个部分组成，一旦与目标靶点结合，即被肿瘤细胞内化。ADC的内化触发ADC各组分分离，在肿瘤细胞中释放出药物，从而诱导细胞死亡^[45]^。hRS7是一种人源化的抗Trop-2抗体，后者广泛表达于多种肿瘤和某些正常组织；而SN-38是伊立替康的活性代谢物，是比伊立替康更为强效的拓扑异构酶Ⅰ抑制剂。两者偶联而成的ADC——IMMU-132在晚期NSCLC患者的Ⅰ期-Ⅱ期临床试验中初步显示了令人肯定的疗效。在既往接受多次治疗（中位三线治疗）的晚期NSCLC患者中，经过确认的客观缓解率达13%，缓解维持时间为9个月，且耐受良好^[46]^。ABBV-399是靶向作用于c-Met的ADC，在体内释放能结合微管蛋白的单甲基耳抑素肽E（monomethyl auristatin E, MMAE），通过抑制有丝分裂达到阻断肿瘤细胞生长的目的。Ⅰ期临床试验显示，该药物用于c-Met表达阳性的NSCLC患者耐受性良好，19%的患者获得部分缓解^[47]^。上述结果还有待在更多的Ⅱ期-Ⅲ期临床试验中进一步验证。此外，多个通过间皮素（间皮瘤、卵巢癌和部分鳞状细胞癌细胞表面高表达；NCT02341625, NCT02485119, NCT02751918, NCT01439152, NCT02839681）、CD30^[48]^（在霍奇金淋巴瘤和非霍奇金淋巴瘤等血液恶性肿瘤细胞表面高表达）和Glypican-3^[49]^（在肝细胞癌、非小细胞肺鳞癌和黑色素瘤等肿瘤细胞表面高表达）等途径的ADC正在接受临床前和临床研究评估。

与ADC类似的是肿瘤靶向放射性核素治疗（targeted radionuclide therapy, TRT），即通过在肿瘤中自然聚集或与肿瘤靶向结合的载体（包括肿瘤特异性抗体），将有细胞毒作用的放射性同位素传递至肿瘤细胞。与ADC相比，TRT的优势在于α和β粒子有一定的作用范围，因此无需内化过程。临床应用的TRT包括^90^Y-替伊莫单抗、^131^I-利妥昔单抗，与其他药物联合用于非霍奇金淋巴瘤患者有较好的效果，其他在研阶段的TCT包括^213^Bi-林妥珠单抗、^225^Ac-林妥珠单抗、^111^In-曲妥珠单抗等^[50]^。

## 3 总结和展望

肿瘤免疫是一个极为复杂的体系，包括一系列活化和抑制信号通路，随着对这些通路的研究逐渐深入，已经发现越来越多潜在有治疗作用的靶点。相关药物的有效性和安全性，包括应用靶向作用于免疫过程中不同环节的药物联合治疗的潜在获益还有待在更多临床研究中进一步明确。与当今国际上日益强调的“精准医学”原则相符的是，未来癌症患者的免疫肿瘤治疗也应强调“精准免疫学”，即确定每例患者肿瘤免疫过程中的缺陷，予以最精确的免疫治疗，将有望改善所有癌症患者的预后。但采用何种生物标志物精准选择靶向治疗人群仍是免疫治疗临床应用中有待解决的问题，需要在更大范围的临床研究中进一步探索。
